# Structural Insights into APOBEC3-Mediated Lentiviral Restriction

**DOI:** 10.3390/v12060587

**Published:** 2020-05-27

**Authors:** Krista A. Delviks-Frankenberry, Belete A. Desimmie, Vinay K. Pathak

**Affiliations:** Viral Mutation Section, HIV Dynamics and Replication Program, National Cancer Institute at Frederick, Frederick, MD 21702, USA; frankenk@mail.nih.gov (K.A.D.-F.); belete.desimmie@nih.gov (B.A.D.)

**Keywords:** APOBEC3G, cytidine deamination, restriction factor, Vif, substrate selection, hypermutation, nucleic acid binding, proteasomal degradation, cullin 5, elongin b and c, CBFβ

## Abstract

Mammals have developed clever adaptive and innate immune defense mechanisms to protect against invading bacterial and viral pathogens. Human innate immunity is continuously evolving to expand the repertoire of restriction factors and one such family of intrinsic restriction factors is the APOBEC3 (A3) family of cytidine deaminases. The coordinated expression of seven members of the A3 family of cytidine deaminases provides intrinsic immunity against numerous foreign infectious agents and protects the host from exogenous retroviruses and endogenous retroelements. Four members of the A3 proteins—A3G, A3F, A3H, and A3D—restrict HIV-1 in the absence of virion infectivity factor (Vif); their incorporation into progeny virions is a prerequisite for cytidine deaminase-dependent and -independent activities that inhibit viral replication in the host target cell. HIV-1 encodes Vif, an accessory protein that antagonizes A3 proteins by targeting them for polyubiquitination and subsequent proteasomal degradation in the virus producing cells. In this review, we summarize our current understanding of the role of human A3 proteins as barriers against HIV-1 infection, how Vif overcomes their antiviral activity, and highlight recent structural and functional insights into A3-mediated restriction of lentiviruses.

## 1. Introduction

To fight against invading bacterial and viral pathogens, eukaryotic organisms have developed not only effective cellular innate and adaptive immunity such as humoral and T cell-mediated immune responses, but also intrinsic immunity, whereby expression of endogenous host restriction factors provides a first line of cellular defense against infections. One important family of mammalian restriction factors that inhibit a variety of viral infections is the apolipoprotein B messenger RNA (mRNA)-editing enzyme catalytic polypeptide (APOBEC) family of proteins [1,2,3]. APOBECs are polynucleotide cytidine deaminases that convert deoxycytidines in foreign DNA substrates to deoxyuridines (dC-to-dU) and/or cytidines in RNA to uridines (C-to-U) [1].

**Figure 1:**
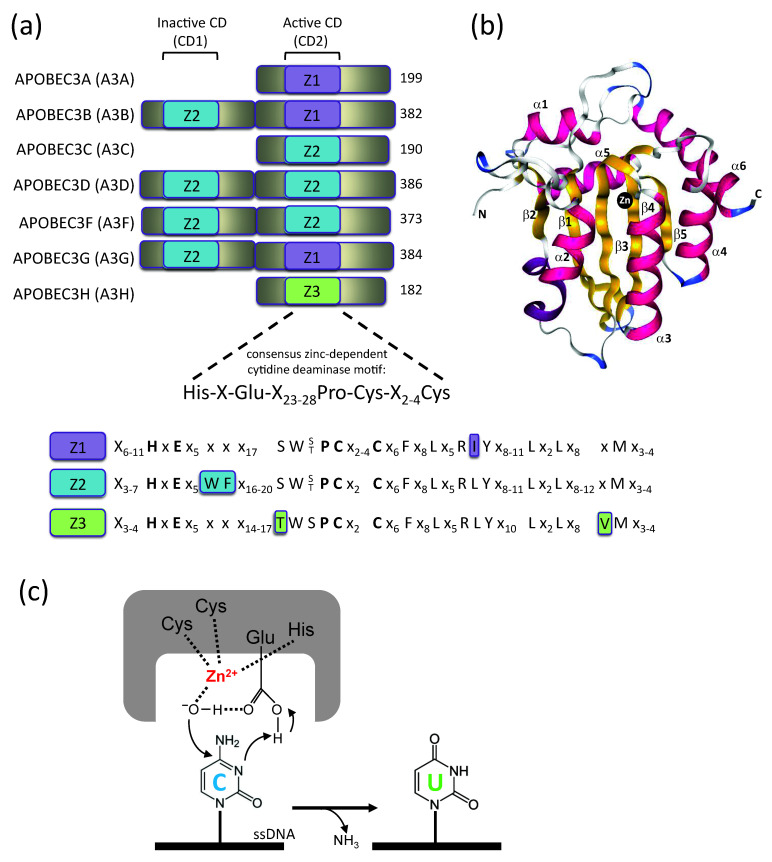
A3 domain organization. (**a**) Zinc-coordinating domains of A3A/B/C/D/F/G/H. A3 gene duplications have given rise to A3A/C/H with single Z-domains and A3B/D/F/G with two Z-domains. Numbers correspond to amino acid sequence length for each A3. Both the catalytically inactive and active Z domains contain the consensus motif H-X-E-X_23-28_-P-C-X_2-4_-C, which is structurally defined as starting at the loop3/α-helix 2 junction. The amino acid similarity between the three Z domains (Z1, Z2, Z3) is shown, each with their distinctive amino acids highlighted. X represents any amino acid. (**b**) Crystal structure of A3 cytidine deaminase (CD) domain. Five β strands (yellow), six α helices (pink), and zinc atom (black) of the A3G catalytic domain are shown (PDB: 3IR2). (**c**) Mechanism of cytidine deamination proceeds by a direct nucleophilic attack on the C4 pyrimidine ring. In the catalytic site, cysteines and histidine together coordinate a zinc atom (Zn^2+^), whereby a water molecule is hydrolyzed, donating a proton to the catalytic glutamate and forming a hydroxyl group. The glutamate acts as a proton shuffle during the catalysis, converting cytidine to uridine.

There are 11 members of the human APOBEC family of genes, which arose by gene duplication, including activation-induced cytidine deaminase (AID), APOBEC1 (A1), APOBEC2 (A2), seven APOBEC3s (hereafter referred to as A3 proteins or A3A, A3B, A3C, A3D, A3F, A3G, A3H), and APOBEC4 (A4) [4]. AID plays a significant role in somatic hypermutation and antibody diversification by class-switch recombination of immunoglobulin genes [5,6]. A1 is responsible for C-to-U editing of the apolipoprotein B transcript at position C6666 and changing a glutamine codon (CAA) to a (UAA) translational stop codon. The cytidine deamination results in the synthesis of the truncated apoB-48 protein, which is important for absorption and transportation of dietary lipid from the small intestine to the liver [7,8]. Currently, the functions of A2 and A4 are still unknown [9,10]. The seven homologous members of the A3 family of proteins all encode a conserved zinc-dependent cytidine deaminase domain (CD) with A3A, A3C, and A3H containing a single cytidine deaminase domain (CD) and A3B, A3D, A3F, and A3G containing a catalytically inactive (CD1) and a catalytically active (CD2) cytidine deaminase domain [4] (Figure 1a). The CDs are organized into three distinct groups based on their homologies, named zinc-coordinating domains Z1, Z2, and Z3 [11,12]. The N-terminal CD1 of A3 proteins A3B, A3D A3F, and A3G are catalytically inactive and function to bind RNA and ssDNA [13,14,15]; they also play a role in the oligomerization of A3 proteins and facilitate processive movement of the A3 proteins on the template to increase the efficiency of cytidine deamination [16,17,18,19]. In addition to sharing their evolutionary origins, the CDs of the A3 family of proteins share fundamental structural homology (Figure 1b). A3 CD domains are composed of five β strands and six α helices centered around a deaminase core with a zinc co-ordinating atom.

The catalytic mechanism of cytidine deamination is believed to be similar to that of an *E. coli* cytidine deaminase, which is homologous to AID and APOBEC1 [20,21,22,23,24,25]. Each CD domain contains a H-X_1_-E-X_23-28_-P-C-X_2-4_-C motif, where “X” is any amino acid (Figure 1a). During the cytidine deamination reaction, a water molecule is hydrolyzed to donate a proton to the catalytic site glutamate and form a hydroxyl group; the cysteines and histidine coordinate a Zn^2+^ atom and the glutamate at the catalytic site functions to shuttle the proton during the hydrolytic deamination reaction to convert cytidine to uridine [4] (Figure 1c).

**Figure 2:**
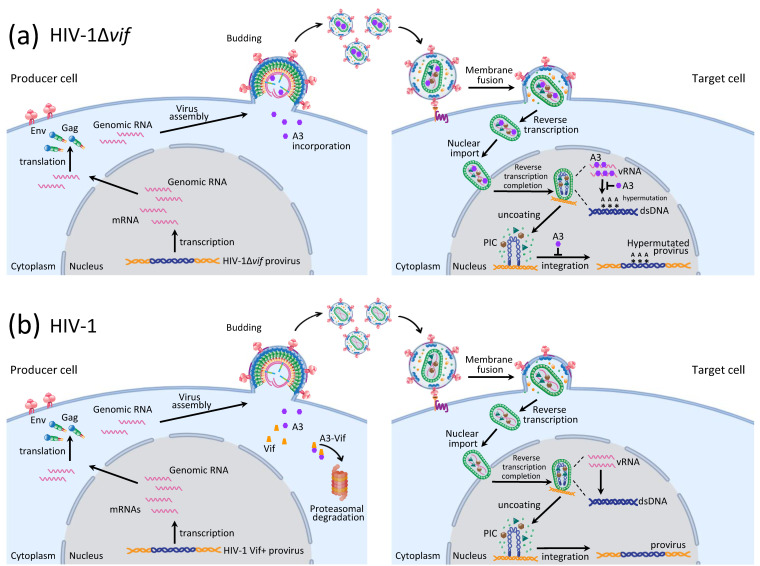
A3 protein-mediated HIV-1 restriction and its counteraction by Vif. (**a**) A3G inhibition of HIV-1Δ*vif*. In the absence of a functional Vif protein (HIV-1Δ*vif*), A3G is packaged into HIV-1 virions in the producer cells, exerting its antiviral activity in the target cells by inducing lethal hypermutation, inhibiting reverse transcription and blocking integration. Reverse transcription and pre-integration complex (PIC) formation occurs within mostly intact viral cores in the cytoplasm and the nucleus [48]. (**b**) Vif induces degradation of A3 proteins. In the presence of wild-type HIV-1, expression of Vif in the producer cells targets A3 proteins for proteasomal degradation, leading to productive HIV-1 infection of target cells. Created with BioRender.com.

A3G, the first member of the A3 family of antiviral factors to be identified, potently inhibits HIV-1 replication in the absence of the virally encoded accessory protein, virion infectivity factor, Vif (Figure 2a) [26]. Subsequently, in the absence of Vif, A3F, A3D, some haplotypes of A3H (haplotypes II, V, and VII), and a haplotype of A3C have been shown to exhibit varying degrees of antiviral activities [27,28]. It is now established that they all induce dC-to-dU deamination in the minus strand DNA of lentiviruses during reverse transcription, which results in G-to-A substitutions in the plus-strand DNA, resulting in missense and stop-codon mutations that block viral replication [29,30,31,32]. This phenomenon of excess G-to-A mutations was initially described in avian spleen necrosis virus (a gammaretrovirus) and referred to as G-to-A hypermutation [33]. The A3 proteins have subsequently also been shown to restrict replication of endogenous retroelements [34,35], other gammaretroviruses (murine leukemia virus), betaretroviruses (mouse mammary tumor virus), alpha-retroviruses (Rous sarcoma virus), spumaviruses (foamy virus), and delta-retroviruses (human T cell leukemia virus type 1) [36,37,38,39,40,41,42,43], as well as adeno-associated virus [44], hepatitis B virus [45], and dsDNA viruses (human papilloma virus, herpes simplex virus type 1) [46,47].

HIV-1 overcomes A3 protein restriction by encoding and expressing Vif, which targets A3 proteins for polyubiquitination and proteasomal degradation, thereby rescuing HIV-1 replication and infection (Figure 2b) [26,49,50,51,52,53,54,55,56,57,58,59]. Vif recruits A3 proteins to the cellular E3 ubiquitin ligase complex composed of cullin 5 (Cul5), elongin b and elongin c complex (EloB/C), and a RING-box subunit 2 (RBX2) to form a CRL5 E3 ubiquitin ligase which polyubiquitinates the A3 proteins, resulting in their proteasomal degradation [50,52,54].

A3G is the most potent inhibitor of HIV-1Δ*vif*; however, A3F, haplotypes II, V, and VII of A3H, and to a lesser extent, A3D can also hypermutate HIV-1Δ*vif* and exhibit less potent but significant antiviral activity [31,57,60,61]. A3G, A3F, A3D, and A3H (haplotype II) are predominantly cytoplasmic proteins (compared to A3A, A3B, and A3H haplotype I, which are predominantly nuclear) that are incorporated into newly formed HIV-1Δ*vif* virions through non-specific interactions with viral genomic RNA or non-viral RNAs such as 7SL RNA [14,62,63,64,65,66,67]. It is estimated that on average, about 7 ± 4 molecules of A3G are incorporated per virion produced from primary CD4^+^ T cells [68]. It is only during reverse transcription in the newly infected target cells that the A3 proteins deaminate (dC-to-dU) the viral minus-strand DNA, leading to lethal G-to-A hypermutation in the complementary plus strand [30,31,69,70,71,72].

It has been proposed that hypermutation by A3 proteins can increase genetic variation in HIV-1 populations and can promote viral evolution [73,74,75,76,77]. In support of this notion, several studies have suggested that resistance to antiviral drugs is increased when A3 proteins are present and can induce G-to-A mutations in the viral genome [78,79,80,81,82]. However, other studies have suggested that A3G-induced hypermutation is an “all or nothing” phenomenon; virion incorporation of A3G and A3F leads to hypermutation and lethal mutational loads which inactivate the provirus and hence do not significantly contribute to viral genetic diversification [80,83,84,85]. This is primarily because the high levels of G-to-A mutations ensure that the proportion of viral genomes that escape termination codon mutations is vanishingly small (4 × 10^−21^ genomes for A3G-induced hypermutation and 1 × 10^−11^ genomes for A3F-induced hypermutation) [84]. In addition, selection against deleterious mutations results in a gradient of hypermutations [86], whereby viral DNAs contain the highest frequency of mutations, mRNAs contain intermediate frequency of mutations, and viral RNAs contain the lowest frequency of mutations. This gradient of hypermutations is the result of the G-to-A mutations having deleterious effects on multiple stages of viral replication, including virus production, transcription, mRNA stability, nuclear-cytoplasmic transport, translation, and virion assembly, which ensures that only those viral genomes with low levels of G-to-A mutations are packaged into virions. Modeling of recombination between a hypermutated genome and a wild-type genome indicated that very little or no hypermutated portions of the genome without lethal mutations can be rescued by recombination to contribute to the genetic diversity of the replicating viral population [84].

In addition to G-to-A hypermutation, A3 proteins can also block viral replication through deaminase-independent mechanisms [87,88,89,90,91,92,93,94,95,96]. A3 proteins can inhibit reverse transcription and reduce viral DNA synthesis indirectly by binding viral RNA and blocking the processivity of reverse transcriptase [87,88,89,90,91,92] or directly by binding to reverse transcriptase (RT) [95]. Furthermore, A3 proteins can also block 3′-processing of the viral DNA and integration into the human genome, but to find out whether this is due to an indirect mechanism that involves A3F binding to viral DNA ends and blocking access to integrase or through direct interactions with integrase requires further studies [93,94,96]. Virion incorporation of A3 proteins in experimental settings can greatly exceed the amounts of A3 proteins incorporated in virions produced from primary activated CD4^+^ T cells [68] and it is important to evaluate antiviral activities under physiologically relevant levels of A3 protein packaging. Interestingly, different A3 proteins exhibit different degrees of deaminase-independent antiviral activities. Introducing a catalytic-site mutation in A3G (E259Q) severely reduces its antiviral activity [93]; in contrast, a similar catalytic site mutation in A3F (E251Q) has only a minimal effect on its antiviral activity [94]. This suggests that A3G largely induces viral inhibition through deaminase-dependent mechanisms, whereas A3F exerts its antiviral effects mainly through deaminase-independent mechanisms (affecting reverse transcription, 3′-processing and/or integration) [93,94]. A3H has also been reported to mainly inhibit viral replication through a cytidine deamination-independent mechanism as little to no G-to-A hypermutation was observed, even with reductions in viral infectivity [97]. In support of this conclusion, it was observed that when A3F and A3H hap II inhibit virus infectivity to a similar extent, a smaller proportion of viral genomes are hypermutated in the presence of A3H compared to A3F and the viral genomes hypermutated by A3H exhibit 3-fold less G-to-A mutations/clone [31].

Human A3 proteins are among the most potent inhibitors of HIV-1 replication in the absence of Vif. Understanding their structure and molecular interactions with Vif proteins may provide molecular targets for the development of novel classes of antiviral drugs that can harness the innate immune defenses to inhibit the replication and spread of HIV-1. Determining the molecular details of how A3 proteins interact with their ssDNA substrates and identify the target cytidine for deamination can also provide valuable insights into how these enzymes can be used to generate more effective and nucleotide-specific gene editing tools [98,99,100]. Here, we summarize the current understanding and recent structural insights into how Vif overcomes the antiviral activity of human A3 proteins and mediates restriction of lentiviruses.

## 2. Overview of A3 Protein and Vif Structures

In addition to sharing their evolutionary origins, the CDs of A3 family of proteins share fundamental structural homology (Figure 1b). Numerous structures of A3 proteins that have been determined so far by x-ray crystallography or nuclear magnetic resonance (NMR) [18,101,102,103,104,105,106,107,108,109,110,111,112,113] are listed in Table 1.

Structures of full-length single-deaminase-domain A3 proteins A3A, A3C, and A3H alone or in complex with single-stranded nucleic acids have been determined. In addition, there are many structures of the C-terminal domains (CTDs) containing the catalytically active domains of double-deaminase-domain A3 proteins, A3B-CTD, A3F_CTD_, and A3G_CTD_. Some recent studies have determined the structures of the catalytic domains of the A3G_CTD_ in complex with its ssDNA substrate. In addition, there are a few structures of the RNA-binding N-terminal domains (NTDs) of A3B_NTD_ and A3G_NTD_. Most recently, a double-domain structure of a full-length rhesusA3G chimera was solved, which suggests that the CD1 and CD2 domains can form different packing orientations to support RNA binding and dimerization/multimerization [131]. Two different full-length structures showed a 29° rotation of CD2 relative to CD1, suggesting that their relative orientations might be flexible. A potential role for a positively charged surface that forms through CD1-CD1 dimerization and could be involved in RNA binding is also evident. However, mutating residues in this positively charged interface did not abolish virion packaging, suggesting that the ^126^FW^127^ residues play a more important role in A3G virion incorporation. All of these structures show that the CDs of A3 proteins share a basic structure exhibiting a canonical CD fold that encompasses a 5 β-strand core surrounded by 6 α-helices; the zinc-dependent catalytic site is formed by the α2- and α3-helices and the β3-strand (Figure 1b) [112,138].

It is essential to elucidate how Vif binds to the various A3 proteins and targets them for proteasomal degradation. Furthermore, to achieve a more comprehensive understanding of the antiviral activities of the A3 proteins at the molecular level, determination of the structures of A3 proteins in complex with their ssDNA substrates is critical. Structural and biochemical studies have led to the precise assignments of some of the residues that are involved in ssDNA binding in the different A3 structures obtained in the presence of nucleic acids [109,110,112,125]. These accumulating structural, functional, and biochemical features provide important insights into A3 protein cytidine deaminase activities and substrate specificities as well as their recognition by HIV-1 Vif-containing CRL5 E3 ligase complex [134]. In the next sections, we review what is currently known about interactions of human A3 proteins with HIV-1 Vif and ssDNA.

## 3. Identification of A3 Residues Critical for A3-Vif Interactions through Mutational Analyses

**Figure 3:**
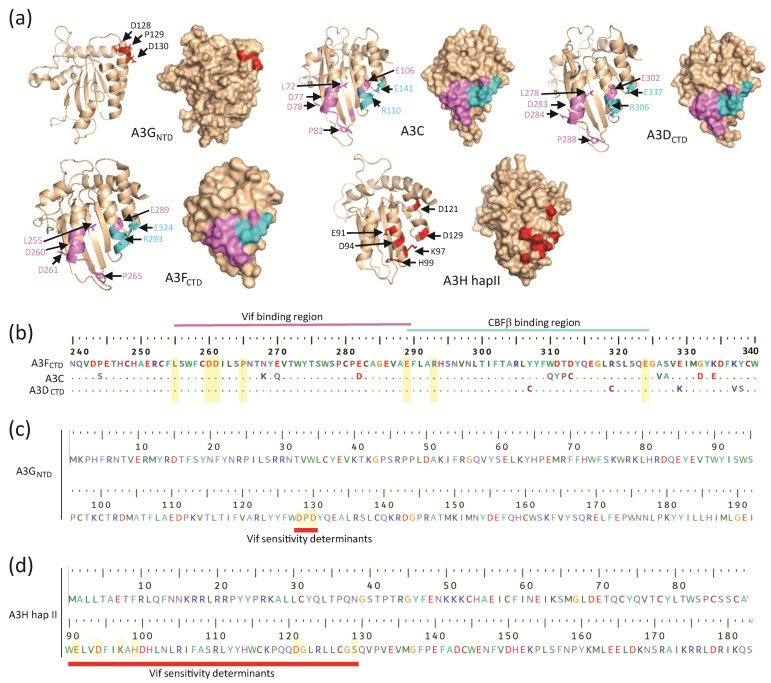
Comparison of A3G_NTD_, A3C, A3D_CTD_, A3F_CTD_, and A3H hap II structures. (**a**) Cartoon and surface representations of the structure of A3G_NTD_ (PDB: 2MZZ), A3C (PDB: 3VOW), and A3D_CTD_ (a predicted model generated through homology modeling based on the A3F_CTD_ structure (PDB: 6NIL) by using the Phyre2 web portal for protein modeling, prediction and analysis [140], and A3H hap II (PDB: 6BBO). The known Vif-mediated degradation determinants labeled in red (for A3G and A3H), the critical residues for Vif-binding in magenta (for A3C, A3D, and A3F), and the CBFβ binding residues in cyan (for A3C, A3D, A3F). (**b**) Multiple sequence alignment of A3 domains (A3F_CTD_, A3C, A3D_CTD_) and the critical Vif- and CBFβ-binding residues are highlighted in yellow bars. A3F was used as a reference and hence the numbering is based on A3F’s residue numbers. The determinants for Vif- and CBFβ-binding are in non-overlapping regions, which are indicated above the alignment. (**c**,**d**) Similar primary sequences of residues in A3G_NTD_ and A3H hap II are shown, respectively. The residues highlighted in yellow bars are identified to be critical for Vif-mediated degradation of the respective A3 proteins. The sequence alignment was performed by a Clustal W multiple alignment in BioEdit.

Until very recently, a structure of any A3 protein in complex with Vif had not been available [123]. Consequently, most of our understanding of structural determinants that are critical for A3-Vif interactions was obtained through mutational analyses of Vif and A3 proteins. These studies identified three putative Vif interaction surfaces that are distinct and are defined as A3G-like, A3F-like, and A3H-like [139]. The A3F-like interaction is very similar to the Vif interaction with A3C or A3D_CTD_. Elucidation of the structures of A3G_CTD_, A3G_NTD_, A3F_CTD_, A3C, and A3H (see references in Table 1) has led to the mapping of existing mutational analysis data onto the solvent-exposed surfaces of the different A3 proteins (Figure 3).

It is important to note that analysis of A3-Vif interactions by mutagenesis and co-immunoprecipitation or A3 degradation assays can only identify the determinants that are functionally important for binding or degradation. Mutational analyses cannot determine whether the amino acids identified as critical residues interact with each other directly or whether they influence binding and A3 degradation through indirect interactions. Indeed, as discussed later, the recently determined structure of an A3F_CTD_-Vif-CBFβ complex clearly showed that some A3F determinants that were predicted to be important for interaction with Vif do not interact with Vif, but instead interact with CBFβ [123].

### 3.1. A3G Determinants That Interact with Vif

Early mutational analyses of human A3G (A3G), guided by amino acid differences with Vif-resistant simian A3Gs, identified A3G-D128 as an essential determinant for Vif-induced degradation and substitution of A3G-D128 with K rendered A3G completely resistant to Vif-mediated degradation [141,142,143,144]. Subsequent studies showed that the ^128^DPD^130^ motif in A3G_NTD_ was critical for Vif-mediated degradation [145]. Letko and colleagues observed that A3G-Y125R substitution mutant was resistant to Vif-mediated degradation, indicating that it was involved in the interaction with Vif [146]. A structure of the A3G_NTD_ showed that the ^128^DPD^130^ residues are exposed on the surface and available to interact with Vif. The residues involved in the Vif interaction form a distinct surface composed of poorly conserved regions in α2, α3, and α4 helices in the A3G_NTD_ (Figure 3a,c). The D128 and D130 residues of the DPD motif and other regions of A3G are under positive selection, which results from host-pathogen genetic conflicts that lead to rapid fixation of mutations altering amino acids at protein-protein interfaces, indicating that the genetic conflict between A3G and Vif has been going on over evolutionary time [147,148].

### 3.2. A3F, A3C, and A3D Determinants That Interact with Vif

Studies of A3G/A3F chimeras showed that in contrast to the interaction of A3G_NTD_ with Vif, the interaction of A3F_CTD_ with Vif is critical for inducing human A3F degradation [149]. Additional mutational analysis of A3F identified ^289^EFLARH^294^ motif as a structural determinant that is essential for Vif binding and A3F degradation [150,151]. Within the ^289^EFLARH^294^ motif, E289 was identified as a single amino acid that is essential for Vif-mediated A3F degradation and an E289K substitution rendered A3F resistant to Vif [150,151]. Another mutational study, guided by sequence differences between Vif-sensitive A3F and Vif-resistant rhesus macaque A3F, identified E324 as a critical amino acid for Vif-mediated A3F degradation [152]. Subsequent determination of the structure of A3F_CTD_ showed that the ^289^EFLARH^294^ motif and E324 are near each other (Figure 3a,b).

The EFLARH motif was conserved in human A3C (A3C; ^106^EFLARH^111^) and human A3D (^302^EFLARH^307^) and substitution of the glutamate in these motifs conferred resistance to HIV-1 Vif-mediated degradation [151]. These results suggested that a similar structural interface in A3F, A3C, and A3D is critical for interaction with HIV-1 Vif.

Determination of a crystal structure of A3C and extensive mutational analyses revealed that a shallow cavity composed of hydrophobic and negatively charged residues in the α2 and α3 helices forms a Vif-interacting surface [106] (Figure 3a,b). Amino acids E106, F107, R110, and H111 are within this interaction surface, confirming a role for the “EF” and “RH” residues of the ELFARH motif in Vif binding. In addition, E141 of A3C, which is equivalent to the E324 residue in A3F, is also part of the interaction interface. Importantly, several additional residues in the α2 helix (L72, F75, C76, I79, L80, S81, Y86) were shown to be critical for Vif binding and mutagenesis of equivalent residues in A3F (L255, F258, C259, I262, L263, S264, Y269) and A3D (L268, F271, C272, I275, L276, S277, Y282) confirmed the importance of these amino acids in A3C, A3D, and A3F for Vif binding [106]. Subsequent biochemical studies have suggested that the L255A, F258A, and L263A substitutions destabilized the A3F_CTD_ structures, which perhaps contributed to their relative resistance to Vif-mediated degradation [120].

### 3.3. A3H Determinants That Interact with Vif

A3H, the only Z3-type cytidine deaminase, has a similar core structure [107,108,133] and the single CD domain is responsible for cytidine deamination, binding to Vif, and binding to RNA. The A3H gene is polymorphic in humans and there are at least seven distinct A3H haplotypes (hap I to hap VII); the mRNAs transcribed from the A3H haplotypes have varying degrees of intracellular stability, resulting in different steady-state levels of A3H proteins and thus, anti-viral activity [153,154,155,156,157]. Of the seven haplotypes, hap II, V, and VII express higher steady-state levels of mRNAs and therefore, proteins that exhibit potent restriction against HIV-1 and LINE-1 retrotransposons. Zhen et al. found that the D121 amino acid in A3H hap II was critical for Vif binding and Vif-induced A3H degradation [158]. Subsequently, extensive mutational analysis by Nakashima and others identified 9 A3H amino acids (S86, W90, V93, D94, I96, K97, D100, D121, L125, S129) that cluster on the surface of helices α3 and α4 that were critical for Vif binding [159,160]. Recently, a 2.49 Å crystal structure of A3H hap II revealed a uniquely long C-terminal helix 6 and a disrupted β5 strand of the canonical five-stranded β-sheet core [132] (Figure 3a,d). Furthermore, this study showed that A3H has a highly positively charged surface, which facilitates RNA-mediated dimers, inhibits its deaminase activity, and modulates its subcellular localization between the nucleus and cytosol. Future structural studies are warranted to determine how the residues identified by mutational analysis dictate the physical interactions between Vif and A3H.

## 4. Identification of HIV-1 Vif Residues Critical for Interactions with the CRL5 E3 Ubiquitin Ligase, A3 Proteins, and CBFβ through Mutational Analyses

### 4.1. HIV-1 Vif Determinants That Interact with CRL5 E3 Ubiquitin Ligase

**Figure 4:**
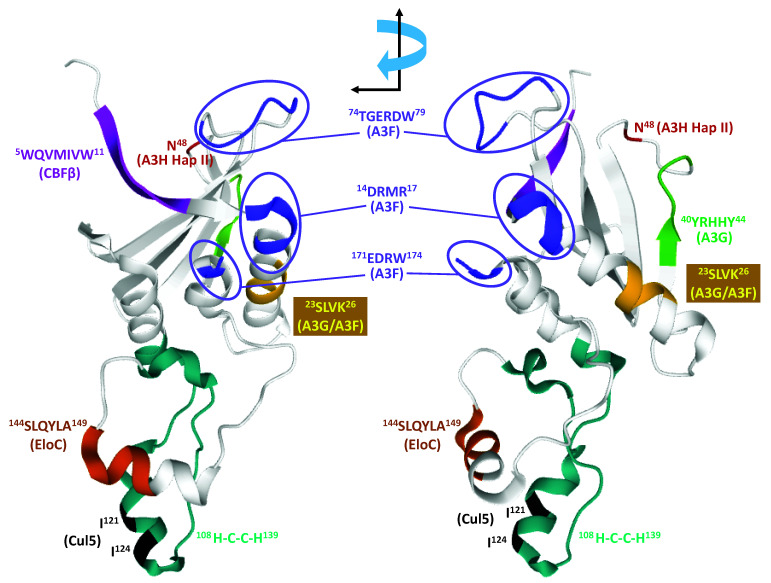
Domains of Vif involved in interactions with A3 proteins and CRL5 E3 ubiquitin ligase. Only the structure of Vif, in complex with CBFβ, Cul5, and EloB/C, is shown (PDB: 4N9F). Residues involved in interactions with A3 proteins are shown using Rasmol software (www.rasmol.org). Residues involved in interaction with A3F, A3G, and A3H hap II are shown in purple, green, and red, respectively. Residues that are involved in interaction with both A3G and A3F are shown in gold. Residues involved in interactions with CBFβ, EloC, and Cul5 are shown in magenta, brown, and black, respectively.

HIV-1 targets A3 proteins for proteasomal degradation by recruiting the A3 proteins to the cellular E3 ubiquitin ligase complex composed of Cul5, elongin b (EloB), elongin c (EloC), and a RING-box subunit (RBX2) complex, resulting in polyubiquitination of the A3 proteins. Soon after the identification of A3G as a host restriction factor [26], it was shown that Vif targets A3G for proteasomal degradation by interacting with cullin 5 and the CRL5 E3 ubiquitin ligase [50,51,52,53,54,161]. Conservation of the ^144^SLQYLA^149^ motif in Vif and human proteins [54,136,162,163] that interact with elongin C identified this region as a critical determinant for interaction with elongin C protein of the elongin B/C (ELoBC) complex (Figure 4). Similarly, the highly conserved ^108^H-X^5^-CX^17-18^-CX^3-5^-H^139^ zinc-binding domain was shown to be essential for interaction with Cul5 [164,165,166]. In addition, amino acids 25–30 in Vif [167] and residues ^120^IR^121^ within the Vif ^108^H-C-C-H^139^ motif [168] were suggested to play a critical role in Cul5 binding (Figure 4).

### 4.2. HIV-1 Vif Determinants That Interact with A3 Proteins

Alanine-scanning mutational analysis of the first 60 amino acids of Vif identified two distinct regions that were critical for Vif-mediated degradation of A3G and A3F [169]. A Vif mutant in which the ^40^YRHHY^44^ region is replaced with alanines is fully capable of degrading A3F but not A3G (Figure 4). Conversely, a Vif mutant in which the ^14^DRMR^17^ residues are substituted with alanines is fully capable of degrading A3G but not A3F (Figure 4). The specificity of these mutants towards A3G and A3F was confirmed in vivo by infection of humanized mice and comparing the patterns of G-to-A hypermutations [170]. In view of these results, it is very interesting that when the ^14^DRMR^17^ is substituted with SERQ or SEMQ (equivalent residues in SIV African green monkey (agm) Vif), it confers the ability to induce degradation of agmA3G as well as the otherwise resistant human A3G-D128K [169], an observation that has been independently confirmed [169]. These observed differences, which were thought to indicate species-specific interactions, were attributed to the two positively charged amino acids (R15 and R17) in HIV-1 Vif and their interactions with the negatively charged A3G-D128. One hypothesis is that the substitution of DRMR with SEMQ creates a new motif that interacts with A3G-D128 in a manner that is distinct from the interaction of wild-type Vif with A3G. However, a structure of a Vif-A3G complex is needed to resolve this mystery.

In addition to the ^14^DRMR^17^ motif, some amino acids in the ^74^TGERDW^79^ motif and the ^171^EDRW^174^ motif were also found to be essential for Vif-mediated degradation of A3F but not A3G [119,171] (Figure 4). Specifically, E76A and W79 in the ^74^TGERDW^79^ motif and all four amino acids in the ^171^EDRW^174^ were essential for Vif-mediated degradation of A3F [119]. Finally, the ^23^SLVK^26^ region was shown to be critical for degradation of both A3G and A3F [172,173].

As discussed earlier, the conserved EFLARH motif is essential for degradation of A3F, A3C, and A3D [151]. Thus, as expected, similar Vif determinants are involved in degradation of both A3F and A3C, but there were some differences in the Vif-A3F and Vif-A3C interactions [119]. Vif amino acids R17, E171, and R173 were much more critical for the Vif-A3F interaction than the Vif-A3C interaction.

HIV-1 Vif uses a distinct motif to counteract A3H hap II compared to the motifs involved in degradation of A3G, A3F, A3C, and A3D. Ooms et al. [174] found that the amino acid variation at position 48 in Vifs dictates the differential ability to induce A3H hap II degradation (Figure 4). Approximately 24.7% of Vifs, including NL4-3 Vif, contain N48 and cannot neutralize the antiviral activity of A3H hap II, whereas 74.1% of Vifs, including the LAI Vif, contain H48 and can efficiently induce A3H hap II degradation (https://hivmut.org). Subsequently, the same group identified that the presence of F39 and H48 substitution in HIV-1 Vif affected the activity against A3H hap II but not against A3G and A3F [175].

### 4.3. HIV-1 Vif Determinants That Interact with CBFβ

In 2011, Jager et al. and Zhang et al. independently identified core binding factor β (CBFβ) as a host factor that specifically binds to HIV-1 Vif and facilitates efficient degradation of A3 proteins [176,177]. CBFβ is a non-DNA binding subunit of the RUNX1, RUNX2, and RUNX3 family of heterodimeric transcription factors that regulates their ability to promote transcription of immunity related genes [178,179,180,181]. Interestingly, the Vif-CBFβ interaction overlaps with the CBFβ-RUNX interaction, which indicates that Vif and RUNX1 are mutually exclusive for CBFβ-binding. The Vif-CBFβ interaction increases the steady-state levels of Vif by preventing its degradation [176,177,182,183] as well as by increasing its biosynthesis [184]. In addition to increasing the stability of Vif, the Vif-CBFβ interaction was shown to sequester CBFβ in the cytoplasm and reduce its ability to promote transcription of genes controlled by the RUNX complexes, which includes A3 genes [185]. Thus, the Vif-CBFβ interaction inhibits A3 protein expression.

Guo et al. [134] determined the structure of a pentameric complex composed of Cul5, EloB/C, Vif, and CBFβ (discussed below), indicating that Vif is tightly associated with CBFβ. Mutational analyses identified several residues of Vif that are important for its in vivo interaction with CBFβ [186,187,188]. Double-alanine scanning mutagenesis of the first 60 amino acids of Vif provided a comprehensive view of Vif determinants essential for in vivo interaction with CBFβ and identified the N-terminal ^5^WQVMIVW^11^ as the major interaction determinant [189] (Figure 4). Furthermore, in agreement with a previous study [190], CBFβ amino acid F68 played a key role in forming a tripartite hydrophobic interaction with CBFβ I55 and Vif W5 to maintain a stable and functional Vif-CBFβ complex. The pentameric structure [134] showed the extensive hydrophobic interactions between the N-terminal anti-parallel β-strands of Vif and CBFβ, which include the ^5^WQVMIVW^11^ region of Vif, and I55 and F68 of CBFβ.

## 5. Structure of the Pentameric Complex of Vif, Cul5, EloB/C, and CBFβ

In a landmark study, Guo et al. [134] determined the structure of a Vif-Cul5-EloB/C-CBFβ pentamer, providing the first structure of full-length Vif (amino acids 1-192) as well as new insights into its interactions with the E3 ubiquitin ligase complex substrate adaptors EloB (residues 1-102) EloC (residues 1-102), scaffold protein Cul5 (residues 12-386), and CBFβ (residues 17-112). The Vif BC-box motif, which contains the conserved ^144^SLQYLA^149^ region that is homologous to the suppressor of cytokine signaling (SOCS)-box motif in SOCS-box proteins, interacts with EloC [191]. The H^108^-C^114^-C^133^-H^139^ zinc-binding motif, which is essential for binding to Cul5, is bound to a Zn^+2^ atom that is solvent inaccessible. Vif residues 116-131 within the H^108^-C^114^-C^133^-H^139^ motif act as a cullin box and mutations of Vif I120S and L124S impaired the Vif-Cul5 interaction (Figure 4). Cul5 residues L52 and W53 play a critical role in binding to Vif.

The Vif-CBFβ complex plays a key role in organizing the pentameric complex and in its absence, interactions between Cul5 and EloB/C were reduced. The importance of Vif residues ^5^WQVMIVW^11^ and CBFβ amino acids I55 and F68 is consistent with the close interactions of these amino acids in the structure. Furthermore, the C-terminal CBFβ helix 5 residues (e.g., F143) also form critical interactions with Vif residues W89, T96, and L106. Some of these residues were previously identified as being critical for interaction with Vif [192] and others have been shown to be critical for the Vif-CBFβ interaction [187,193]. Importantly, the larger buried surface area of the Vif-CBFβ interaction compared to the CBFβ-RUNX1 interaction (4797 Å vs. 3941 Å) suggests that CBFβ has a higher affinity for Vif than RUNX1. The suggestion that Vif evolved a stronger affinity to CBFβ than its cellular partner RUNX1 underscores the importance of the Vif-CBFβ interaction for its role in overcoming restriction by A3 proteins.

Overall, the pentameric structure indicated that the Vif interacting proteins Cul5, EloC, and CBFβ interact with hydrophobic portions of Vif and leave the positively charged surfaces free to interact with A3 proteins. Most of the Vif amino acids previously identified to be critical for interactions with A3 proteins are exposed on the Vif surface and available to interact with A3 proteins (see Figure 4).

## 6. Structure of a Vif-CBFβ-A3F_CTD_ Ternary Complex

**Figure 5:**
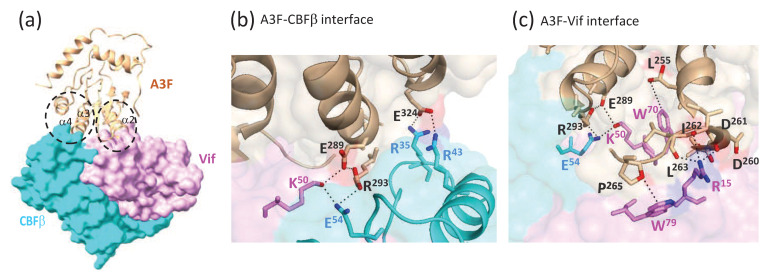
Ternary A3F_CTD_-Vif-CBFβ complex and the role of CBFβ-Vif dimer in recruiting A3F_CTD_ to the CRL5 E3 ubiquitin ligase complex. (**a**) Overall structure of A3F_CTD_-Vif–CBFβ ternary complex. Vif (magenta) and CBFβ (cyan) form a shallow wedge-like platform for A3F_CTD_ (orange) to dock primarily via the α2-helix on Vif and the α3- and α4-helical surface on CBFβ. (**b**) Detailed illustrations of the A3F-CBFβ interface and interacting residues that form two distinct electrostatic interfaces; the first involves A3F (E289 and R293), CBFβ (E54), and Vif (K50); the second involves A3F (E324) and CBFβ (E35/E43). (**c**) Detailed illustrations of the A3F-Vif interface and interacting residues. The extensive hydrophobic and electrostatic A3F-Vif interactions are indicated with dashed lines and all critical residues involved in the interactions are shown in stick form.

A cryogenic electron microscopy (cryo-EM) structure of a ternary complex consisting of Vif, CBFβ, and A3F_CTD_ at 3.9 Å resolution was recently reported [123], providing the first structure of a Vif-A3 complex. As discussed earlier, the Vif-CBFβ interaction was thought to facilitate Vif-mediated degradation of A3 proteins by primarily inhibiting degradation of Vif and increasing its steady-state levels. Furthermore, it was thought that Vif binding to CBFβ partially blocks A3 binding and that CBFβ must be displaced before Vif can bind to A3 proteins [139,186]. It was therefore surprising that binding to A3F_CTD_ did not significantly alter the conformation of the Vif-CBFβ complex. Additionally, it was found that CBFβ directly interacts with A3F_CTD_ and participates in the recruitment of A3F_CTD_ to the Cul5 E3 ubiquitin ligase complex (Figure 5a).

The structure shows that Vif and CBFβ form a stable platform to which A3F_CTD_ binds. A3F_CTD_ binding does not induce significant conformational changes in the Vif-CBFβ complex or in the A3F_CTD_. Local conformational changes in the Vif loop between β4 and β5, which include the ^23^SLVK^26^ region previously shown to be important for A3G and A3F binding [172,173], brings this loop closer to the Vif C-terminal residues 173–176, which overlap the ^171^ERDW^174^ region previously shown to be important for A3F binding [119,192].

The structure revealed the CBFβ-A3F_CTD_ interface and the Vif-A3F_CTD_ interface (Figure 5b,c). The importance of the CBFβ-A3F_CTD_ interface in vivo was established through mutational analysis of critical CBFβ and A3F_CTD_ residues at the interface. CBFβ residues R35 and R43 form a positively charged surface that interacts with a negatively charged surface on A3F_CTD_ composed of E324 and several main chain carbonyls. The negatively charged CBFβ residue E54 interacts with the positively charged A3F residue R293, which is stabilized by Vif H73. A3F R293D mutant was resistant to degradation by Vif and CBFβ E54K mutant interfered with WT Vif’s ability to degrade A3F. Remarkably, the A3F mutant R293D became sensitive to Vif-mediated degradation in the presence of the CBFβ mutant E54K. These charge-swapped pairs of mutants reversed the Vif-resistance phenotype in vivo, indicating that A3F R293 and CBFβ E54 physically interact.

The physiological relevance of the Vif-A3F interface was also determined by mutational analyses of Vif and A3F_CTD_ residues predicted to be critical for the interaction. Three major Vif-A3F_CTD_ interactions were identified in the structure. First, Vif W79A formed a stacking interaction with A3F P265. The Vif double-mutant W79A-H80A was unable to induce degradation of WT A3F and the A3F mutant P265A was partially resistant to WT Vif, indicating the importance of these amino acids for Vif-mediated A3F degradation. Second, Vif R15 forms a strong electrostatic interaction with the negatively charged main chain carbonyls of A3F residues 260–263. As predicted from previous studies indicating the importance of the ^14^DRMR^17^ motif in Vif [169], the Vif mutants R15D or R15E failed to induce degradation of WT A3F or rescue infectivity in single replication cycle assays. Third, Vif K50 residue, together with CBFβ E54, formed electrostatic interactions with A3F E289 and R293 residues. Vif mutant K50E was severely defective in inducing A3F degradation or rescuing virus infectivity in single cycle assays and as shown previously [151], A3F mutant E289K was highly resistant to degradation by WT Vif.

Overall, the A3F_CTD_-Vif-CBFβ structure provides a comprehensive model of how A3 proteins bind to the Vif-CBFβ dimer and are recruited to the Cul5-EloB/C complex for polyubiquitination and proteasomal degradation. The structure clarifies much of the mutagenesis data; some of the A3F-Vif interactions were confirmed, while other interactions thought to be between Vif and A3F are actually A3F-CBFβ interactions. For example, A3F residues R293 [119,150,151] and E324 [152] were thought to interact with Vif, but actually interact with CBFβ [123]. Other residues that were thought to be involved in Vif-A3F interaction are located in the interior of the structure and indirectly influence Vif-mediated degradation of A3F. These insights reinforce the notion that both structural studies and in vivo mutational analyses are needed to attain a full understanding of the Vif-A3 interactions.

It is also important to note that the interactions of the Vif-CBFβ complex with A3G are likely to be quite different from the interactions with A3F, since the CBFβ mutations that inhibit degradation of A3F (R35E, R43E, E54K) do not inhibit degradation of A3G. Based on the conservation of the EFLARH motif and its importance in Vif-mediated degradation of A3F, A3D, and A3C, the Vif-CBFβ interactions with A3D_CTD_ and A3C are likely to be similar but may well exhibit differences. Thus, it will be important to obtain structures of the other A3 proteins in complex with the Vif-CBFβ complex.

## 7. Insights into Substrate Selection, A3 Deamination, and Editing-Site Selection

Cytosolic A3D/F/G/H proteins are incorporated into newly budding virions through interactions of their N-terminal RNA binding domain with HIV-1 viral RNA, leading to HIV-1 restriction through both editing and non-editing mechanisms. The CTD domain for double domain A3s retains the deaminase and hypermutation activity, while the NTD domain, which is catalytically inactive, retains important functions for nucleic acid binding, oligomerization, packaging, and processivity [13,113,149,194,195,196,197]. Extensive G-to-A hypermutation [32,52,70,83,84] of viral genomes in patients results in an average of ~20% of G residues mutated to A residues during a single round of reverse transcription, leading to lethal mutagenesis and viral inactivation [84]. During reverse transcription as minus-strand synthesis progresses, RNase H degradation of the viral RNA template allows A3G to access the newly synthesized ssDNA (minus-strand DNA), inducing deamination of dC to dU. A3G lacks deaminase activity on dsDNA or RNA templates [71,149,194,198]. The dC-to-dU deaminations on the (-) strand DNA then template G-to-A mutations to the (+) strand DNA as RT completes plus-strand DNA synthesis, forming a dsDNA viral genome.

### 7.1. ssDNA Substrate Selection

**Figure 6:**
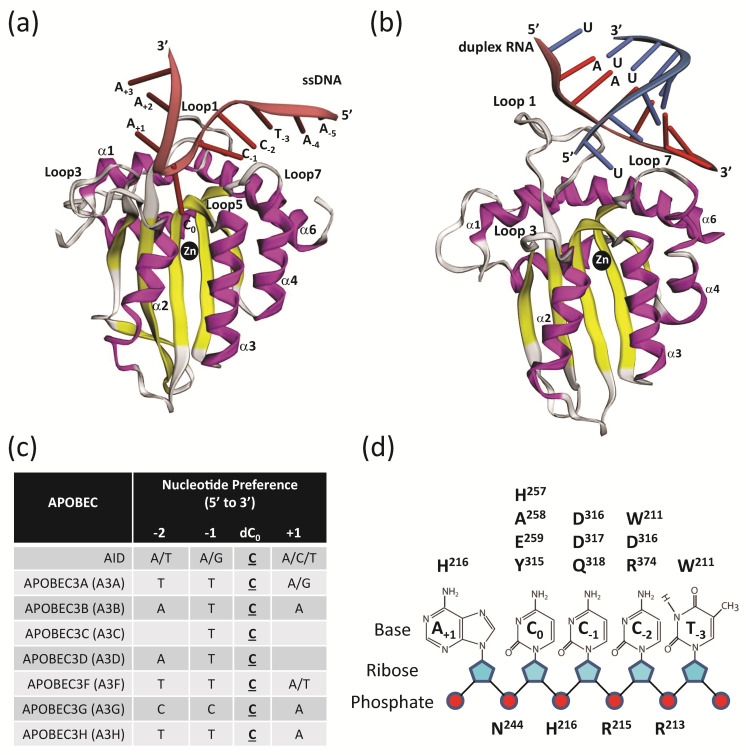
A3 deamination and ssDNA interactions. (**a**) Co-crystal structure of A3G_CTD_ in complex with ssDNA substrate 5′-AATCCCAAA-3′ (PDB 6BUX). Shown is the three-dimensional fold of the A3G_CTD_ with α-helices (purple) and β-sheets (yellow) with the ssDNA (red) co-ordinated with the reactive cytosine (C_0_) positioned next to the catalytic zinc atom. Loops 1, 3, 5, and 7, which influence substrate binding, specificity, and dinucleotide preference selection, are labeled. (**b**) Co-crystal of A3H in complex with duplex RNA substrate 5′-UAAAAAAA-3′ + 5′-UUUUUUUUU-3′ with a 1 nucleotide overhang (PDB 6B0B). Shown are α-helices (purple) and β-sheets (yellow) with the duplex RNA (red and blue) positioned with zinc atom. (**c**) Preferred ssDNA substrates of the A3 enzymes where the underlined C is the preferred target nucleotide of the deamination reaction. (**d**) Summary of the interactions of the 5′-TCCCA target ssDNA substrate with amino acids in A3G_CTD_ (PBD 6BUX). A3G loop 1 contains amino acids W211, R213, R215, H216; A3G loop 3 contains amino acids N244, H257; A3G α-helix 2 contains amino acids A258 and E259; A3G loop 7 contains amino acids Y315, D316, D317, Q318 and A3G α-helix 6 contains amino acid R374.

Even though A3 proteins share an overall similar architecture with a common conserved catalytic domain (H-x-E-x_25-30_-P-C-x_2-4_-C) comprising six α-helixes and 5 β-sheets, A3G prefers to deaminate ssDNA 5′-CC motifs to 5′-CU, resulting in 5′-GG-to-AG mutations in the viral plus-strand DNA [30,198], while the remaining A3 proteins prefer to deaminate the ssDNA 5′-TC motifs to 5′-TU, resulting in 5′-GA-to-AA mutations in the plus-strand DNA [44,56,59,153,198,199,200,201,202]. Subtle differences in the interaction pocket of the A3 catalytic domain (CTD) with substrate ssDNA, as well as variability in A3 protein loops L1, L3, L5, and L7 proximal to the active site likely influence substrate binding, specificity, and dinucleotide preference selection for cytidine deamination (Figure 6a) (reviewed in [203]).

Structures of the A3 NTD or CTD domains in the presence or absence of nucleic acid have broadened our understanding of A3-ssDNA interactions. Low resolution structures of high molecular mass and low molecular mass full-length A3G that provided an overall shape of the A3G were determined by small-angle X-ray scattering [204]. Structures of the catalytically active domains of A3A, A3B, A3C, A3F, A3G, and A3H were determined in the absence of ssDNA, leading to three historical models of how ssDNA coordinates in the catalytic active site of the A3 proteins [18,101,103,104,105,106,109,110,113,120,126,128,129,130,205,206]. The “brim” model proposed that a ring of positively-charged amino acids which are positioned surrounding the concave catalytic active site aid in positioning the target cytidine (dC_0_) for deamination [101]. Additionally, the “kinked” [18,126] and “straight” models [129,206] proposed that the ssDNA substrate is either in a bent or straight configuration in the CTD active site, respectively. The 3.1 Å crystal structure of A3F_CTD_ predicted a single ssDNA binding groove, leading to the catalytic active site supporting the “straight” model [120], while an NMR structure of A3A modeled the ssDNA substrate with a “kink” or a bend so that the reactive cytidine is positioned into the active site [103].

However, recent co-crystal structures of A3A and A3G complexed with ssDNA have now been solved, providing valuable new insights into A3 substrate binding and specificity. The A3A-ssDNA 2.2 Å co-crystal structure shows the binding groove of a DNA oligonucleotide (5′-TTTTTT**TC**TTTTTTT-3′) bound in the active site of A3A, ready for catalysis centering on the deamination target 5′-TC-3′ with the ssDNA positioned in a U-shaped conformation [110]. Three nucleotide contacts are made with target deoxycytidine and two flanking nucleotides and the A3A amino acid H29 is thought to “latch” and stabilize the substrate ssDNA at the target cytidine (dC_0_). This unique U-shaped DNA conformation is also confirmed by a 3.1 Å co-crystal structure of A3A-ssDNA (5′-AAAAAA**TC**GGGAAA) and a 1.7 Å co-crystal structure of an A3B/A3A-ssDNA chimera with Y315 playing a role in forming an open or closed catalytic cleft for ssDNA binding [109]. Further modeling studies of A3B_CTD_ bound to ssDNA supports this U-shaped conformation [207].

A recent study determined the co-crystal structure of A3G_CTD_ with ssDNA at 1.86 Å resolution that contained its relevant substrate 5′-AA**TCCCA**AA-3′, with the ssDNA adopting a curved shape in the active site and W211 playing a critical role in substrate recognition (Figure 6a) [112]. In this structure the ssDNA has a more extended conformation contacting 5 nucleotides in the CTD instead of three as was observed in the A3A-ssDNA co-crystal. Overall, the ssDNA substrate binding conformation in complex with A3 suggests that this U-bent region of nucleic acid is a hotspot for A3 cytidine deamination and may be common to all A3 proteins. Indeed, the tRNA adenosine deaminase TadA from *Staphylococcus aureus* co-crystal structure in complex with tRNA also shares structural similarity with A3A-ssDNA, suggesting evolutionary conservation [208].

APOBEC proteins have evolved to select different ssDNA substrates, reflecting a diversity of their function. Unlike A3 proteins, AID has evolved to target dsDNA for immunoglobulin class-switch recombination and V(D)J hypermutation. The first co-crystal structure of a maltose-binding protein (MBP)-AID fusion protein and dCMP was published showing potential surface grooves that favored G-quadruplexed DNA substrates over linear ssDNA that likely guide AID substrate recognition and facilitate double-strand breaks for class switch recombination [205]. Here, no evidence of a U-shaped substrate channel was observed, likely reflecting a more rigid nature of the dsDNA substrate. Thus, although all APOBECs have a common cytidine deamination function, the overall protein structure at the catalytic core influences substrate specificity.

Interactions of the ssDNA substrate outside of the core catalytic active site have also been reported to influence substrate selection and cytidine deaminase activity. A co-crystal structure of A3F_CTD_ bound to poly-dT(10) shows a new ssDNA-binding surface distal to the zinc active center mediated by hydrophobic and electrostatic interactions between the ssDNA substrate, tyrosine (Y333), and lysine (K352/K355/K358) residues in A3F_CTD_ [121]. These amino acids were also shown to play a role in RNA binding as well as the catalytic activity of A3F. A 1.9 Å crystal structure of soluble A3B_NTD_ mutant showed two positively-charged amino acid patches around the NTD domain that likely bind nucleic acid through electrostatic interactions and facilitate cytidine deamination [117]. In addition, the positively charged CD1 of A3G has been shown to crosslink ssDNA via mass spectrometry and mutations Y181A/Y182A have reduced deaminase activity [209]. The 2.0 Å co-crystal structure of rhesus A3G-CD1 bound to poly-dT ssDNA shows strong ssDNA-binding affinity, which is likely due to the positively-charged surface of rhesus A3G [113]. Indeed, crystal and solution structures of A3G_CTD_ [101,126] lacking the NTD domain have severely reduced deaminase activity, implicating the importance of regions outside the catalytic domain in influencing substrate selection and deamination. Overall, the negatively charged phosphate backbone of the ssDNA substrate requires stabilization via positively charged patches and grooves along the surface of the A3 protein to mediate deamination [104,128].

Recently, a 3.28 Å crystal structure of human A3H showed that A3H is bound to a short RNA duplex [108] (Figure 6b). A3H proteins purified from *E. coli* were devoid of cytidine deaminase activity unless they were treated with RNase A, suggesting that RNA binding inhibits cytidine deaminase activity. Mutagenesis of residues in loop 7 and α6 helix increased cytidine deamination activity, supporting the notion that RNA binding and deamination activities are separated. A model was proposed in which A3H dimerization is promoted by RNA binding, which facilitates cytoplasmic localization, as previously shown for A3G [210], promotes virion incorporation, and increases deaminase-independent inhibition by binding to the template RNA and indirectly inhibiting reverse transcription. On the other hand, RNA binding and dimerization inhibited the cytidine deamination activity. Interestingly, Bohn et al. reported a 2.24 Å crystal structure of A3H from pig-tailed macaques (pgtA3H), which showed that the pgtA3H also forms a dimer around a short double-stranded RNA [107]. However, in contrast to the human A3H, the pgtA3H was reported to retain potent cytidine deaminase activity while retaining binding to short RNA duplexes in the viral genome. These studies have raised interesting questions regarding the nature of RNA sequences that promote A3H binding, dimerization, virion incorporation, and regulation of cytidine deaminase activity.

### 7.2. Deamination and Editing-Site Selection

The sequence context and secondary structure of the ssDNA as well as A3 protein secondary structure can influence site preferences that determine patterns of cytidine deamination. Studies show that the local nucleotide sequence context surrounding the dC_0_ site of deamination (−1, +1, and +2 nucleotides) influences cytidine deamination frequencies for each of the A3 proteins (Figure 6c) [31,125,198,211,212,213,214,215,216]. Detailed hypermutation studies of the −2 position showed that A3G preferred to mutate 5′-TC or 5′-CC dinucleotides when the −2 nucleotide was a C (5′-CCC-3′; the deaminated C is underlined) [31]. A3F and A3H preferred to mutate TC dinucleotide sites when a T was at the −2 position (5′-TTC-3′) and A3D preferred to mutate TC sites when an A was at the −2 position (5′-ATC-3′). Interestingly, all A3 proteins strongly disfavored a G at the −2 position [5′-G(T/C)C-3′]. The most distantly related APOBEC, AID, exhibits a different pattern of hypermutation, favoring 5′-(A/T)(A/G)C-3′ and disfavoring the A3G hotspot 5′-CCC-3′ [213]. Knowing the patterns of editing-site selection, proviral genomes from infected patients can be analyzed for hypermutation patterns; sequence analysis provides evidence that A3 proteins can copackage and comutate the viral genome [31] to block HIV-1 infection. In this study, co-packaging and the increase in G-to-A hypermutation was additive when A3G and A3F were co-expressed and evidence of a synergistic increase when A3G and A3H hap II were co-expressed. However, another study used a single plasmid expression system to express both A3G and A3F at low levels and observed a modest enhancement of A3G deamination activity by A3F (~1.5 fold) compared to the expected additive effect [217]. This result suggests that at low levels of A3 protein expression, it is possible to observe synergistic cooperativity between A3G and A3F.

Structural and biochemical studies have provided insight into how A3G selects the substrate site for deamination. Similarities exist amongst the A3 proteins, but differences in amino acid sequence, overall size of the protein, as well as sequence and length variation in loops L1, L3, and L7 contribute to the differences in substrate recognition and cytidine deamination patterns [18,103,104,105,218,219]. Loop 7, in particular, amino acids R313, Y315, D316, and D317, have been implicated in structural models to play a role in ssDNA binding to the −1 nucleotide and proper positioning for deamination and notably have been proposed to be a “nucleotide specificity box” [201,218,220,221]. Interestingly, swapping the A3G loop 7 into AID at the complementary position changed the site-selection preference of AID to A3G in in vitro assays on ssDNA substrates [220]. A recent 2.9 Å co-crystal structure of an A3G_CTD_ fusion protein with the substrate hot spot sequence 3′-CCCA-5′ captured the substrate bound to A3G with the non-preferred “A” in the −1 nucleotide position in the binding pocket of A3G [125]. Structural analysis showed that rearrangements in the pocket were not conducive to deamination (no rearrangement of A3G D316 flipping to interact with the −1 nucleotide or DNA binding at dC_0_), showing that the precise conformation of the catalytic binding pocket is needed for selective deamination. Indeed, A3B crystal structures in the presence or absence of ssDNA show that in the absence of ssDNA substrate, loops 1 and 7 formed a closed structure with R211 from loop 1 and Y315 from loop 7 stacked on each other, blocking access to the active site; yet in the presence of ssDNA, the loops underwent a conformational switch to accommodate the ssDNA substrate [109,115,116]. Further biochemical and structural studies have also confirmed the importance of loop 7 in editing site selection [20,145,150,201,222]. Mutation D317Y in A3G loop 7 changed the deamination profile of A3G from 5′-CC to 5′-TC and swapping A3G loop 7 with A3A switches A3G deamination preference to 5′-TC [218]. Mutations in loop 7 of A3F decreased ssDNA substrate binding and deamination activity [223] and swapping ^315^YDDQ^318^ of A3G to ^307^YYFW^310^ of A3F switched the substrate preference of A3G from 5′-CC to 5′-TC or 5′-GC [120]. This was also true for AID when loop regions of AID were swapped with corresponding amino acids of A3G or A3F, which resulted in the swapping of substrate preferences [220,221].

Co-crystal structure of A3G_CTD_ with its preferred ssDNA substrate 5′-AA**TCCCA**AA-3′ (favored sequence shown in bold) at high resolution has provided detailed insights into the interactions of A3G residues and the ssDNA [112] (Figure 6d). The target cytidine (C_0_) base interacts with the ^257^HAE^259^ sequence that defines the zinc-binding cytidine deaminase domain and Y315 (loop 7). The cytidine base at the −1 position (C_−1_) interacts with the ^316^DDQ^318^ sequence in loop 7. The cytidine at the −2 position (C_−2_) interacts with D316 (loop 7) and R374 (α-helix 6). The W211 residue interacts with both the cytidine at the −3 position (C_−2)_ and thymidine at the −3 position (T_−3_). The H216 residue interacts with the adenine at the +1 position (A_+1_). In addition to the interactions with the bases, positively charged residues R213, R215, H216, and N244 interact with the phosphate backbone to neutralize the negative charges. A variety of interactions that include pi-pi interactions, hydrogen bonds, water-mediated hydrogen bonds, and hydrophobic interactions serve to stabilize the binding and position the target cytidine for deamination. Thus, the specificity of editing site selection is driven by many structural factors and interactions.

## 8. Summary

The A3 family of proteins provides potent protection against a wide variety of viruses and intrinsic immunity to the host through deaminase-dependent and deaminase-independent mechanisms of inhibition. Cumulative biochemical and structural knowledge has improved our understanding of how A3 proteins bind to Vif and the recent discovery of A3F binding to CBFβ suggests that CBFβ has a direct role in recruiting A3F, and likely other A3 proteins, to the CRL5 E3 ubiquitin ligases. We are also beginning to gain a structural understanding of how A3 proteins bind to the ssDNA substrate and select the sites of cytidine deamination. Importantly, A3 residues distal to the catalytic site, including residues in the non-catalytic N-terminal domain, can influence the sites of cytidine deamination.

Even with the remarkable advances in our understanding of the structure and function of A3 proteins, important questions remain unanswered. Although the structure of a rhA3G full-length protein was recently determined [131], so far, there is no structure of a full-length human double-deaminase domain A3 protein and we do not know how the two CD domains in human A3 proteins are oriented with respect to each other. Resolving the structure of a full-length A3G and/or A3F could provide vital insights into how these proteins bind to their long ssDNA. We need to obtain structures of other A3 proteins bound to the Vif-CBFβ-CRL5 E3 ubiquitin ligase complex and compare these structures with the Vif-CBFβ-A3F_CTD_ complex to determine how their Vif-A3 interactions differ. Importantly, a structural understanding of how A3 proteins inhibit viral replication through deaminase-independent mechanisms is needed. A thorough structural understanding of the Vif-A3 interactions could facilitate the rational design and development of novel therapeutics in the fight against AIDS.

## Figures and Tables

**Table 1:** Structures of Vif and A3 proteins.

A3 Protein and Vif Structures	PDB ^1^ ID	Resolution (Å) ^2^	Modeled DNA or RNA Substrate Sequence	Vif-Binding Interface	Ref. No.
**A3A**	2M65	NMR			[103]
	4XXO	2.84			[114]
	5KEG	2.2	5′-TTCTT-3′		[110]
	5SWW	3.15	5′-ATCGGG-3′		[109]
**A3B_CTD_**	5TD5 ^3^	1.72	5′-TTCAT-3′		[109]
	2NBQ	NMR			[111]
	5CQD	2.08			[115]
	5CQH	1.73	dCMP		[115]
	5CQI	1.68			[115]
	5CQK	1.88			[115]
	5SXG	1.93			[116]
	5SXH	1.78			[116]
**A3B_NTD_**	5TKM	1.9			[117]
**A3C**	3VM8	3.0		yes	[106]
	3VOW	2.15		yes	[106]
**A3F_CTD_**	5HX5	2.33			[118]
	3WUS	2.54		yes	[119]
	4J4J	3.1		yes	[120]
	5HX4	1.92			[118]
	5W2M	3.7	5′-TTTTTTTTTT-3′		[121]
	5ZVA	2.3	5′-ATTTTCAACT-3′		[122]
	5ZVB	2.0	5′-ATTTTCAAT-3′		[122]
	4IUO	2.75		yes	[105]
**A3F_CTD_-Vif-CBFβ**	6NIL	3.9, cryoEM		yes	[123]
**A3G_CTD_**	6K3J	NMR	5′-ATTCUU^I^AATT-3′		[124]
	6K3K	NMR	5′-ATTCUC^I^AATT-3′		[124]
	6BUX	1.86	5′-AATCCCAAA-3′		[112]
	6BWY	2.9	5′-AGGTTAC-3′		[125]
	4ROV	1.8			[126]
	4ROW	1.7			[126]
	3V4J	2.04			[127]
	3V4K	1.38			[127]
	3IR2	2.25			[104]
	3IQS	2.3			[18]
	2KEM	NMR			[128]
	3E1U	2.3			[18]
	2JYW	NMR			[101]
	2KBO	NMR			[129]
**A3G_NTD_**	2MZZ	NMR			[130]
**rhA3G_NTD_^4^**	5K83	2.39	5′-TTTTTTTTTT-3′		[113]
	5K81	2.0			[113]
	5K82	2.93			[113]
**rhA3G**	6P40	2.47			[131]
**full length**	6P3Z	2.8			[131]
**chimera ^5^**	6P3X	2.4			[131]
	6P3Y	2.5			[131]
**A3H**	5W45	2.49			[132]
	6B0B	3.28	7bp duplex RNA with 1 nt overhang: 5′-UAAAAAAA-3′ + 5′-UUUUUUUUU-3′		[108]
	6BBO	3.43	7-bp duplex RNA with 1 nt overhang: 5′-UAAAAAAA-3′ + 5′-UUUUUUUU-3′		[108]
**pgtA3H ^6^**	5W3V	2.24	7-bp duplex RNA with 2 nt overhang: 5′-AACCCGGGGA-3′ + 5′-AACCCCGGGC-3′		[107]
**cpzA3H ^7^**	5Z98	2.2	9-bp duplex RNA with 1 nt overhang: 5′-CUGCCGGGUA-3′ + 5′-AUACCCGGCA-3′		[133]
**Vif-CBFβ-Cul5-ElOB-ElOC**	4N9F	3.3			[134]
**Vif SOCS-box and ElOBC**	2MA9	NMR			[135]
**Vif BC-box with ElOB**	3DCG	2.4			[136]
**Vif_SIVRCM_-CBFβ-ElOB-ElOC ^8^**	6P59	2.9			[137]

^1^ PDB, Protein Data Bank (https://www.rcsb.org). ^2^ Structures are by X-ray crystallography unless otherwise stated. ^3^ 5TD5 is an A3B_CTD_ chimeric structure containing loop 1 from A3A. ^4^ rhA3G is rhesus macaque A3G. ^5^ Chimera of a double-domain rhesus A3G with 8 residues from the CD1 loop 8 replaced by 4 residues from the loop 8 of human A3G-CD2. ^6^ pgtA3H is pig-tailed macaque A3H. ^7^ cpzA3H is chimpanzee A3H. ^8^ Vif_SIVRCM_ is Vif from Simian Immunodeficiency Virus isolated from red-capped mangabey (SIVrcm).
